# Supplementary material for: Life‐long impairment of glucose homeostasis upon prenatal exposure to psychostimulants
Source: EMBO J. 2019 Nov 21;39(1):e100882. doi: 10.15252/embj.2018100882 (PMC6939201; doi:10.15252/embj.2018100882)
Supplement: Supplementary file 1 — Appendix [file EMBJ-39-e100882-s001.pdf]

Appendix to:

Life-long impairment of glucose homeostasis upon prenatal exposure to psychostimulants

Solomiia Korchynska[+], Maria Krassnitzer[+], Katarzyna Malenczyk[+], Rashmi B. Prasad, Evgenii O. Tretiakov, Sabah Rehman, Valentina Cinquina, Victoria Gernedl, Matthias Farlik, Julian Petersen, Sophia Hannes, Julia Schachenhofer, Sonali N. Reisinger, Alice Zambon, Olof Asplund, Isabella Artner, Erik Keimpema, Gert Lubec, Jan Mulder, Christoph Bock, Daniela D. Pollak, Roman A. Romanov, Christian Pifl, Leif Groop, Tomas G.M. Hökfelt & Tibor Harkany*

[+]S.K., M.K. and K.M. contributed equally to this study.

This file contains:

Appendix Figures S1-S6 and their Legends,

References.

Appendix Figure S1

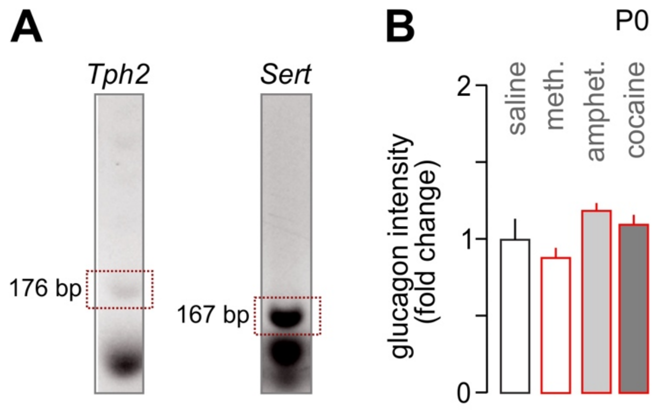

Determinants of psychostimulant sensitivity in pancreatic islets.

(**A**) Reverse-transcription PCR products for *Tph2* and *Sert* in E14.5 mouse pancreata. (**B**) Prenatal drug exposure does not affect glucagon immunoreactivity at birth (P0). Data from triplicate experiments shown as means ± s.d.

Appendix Figure S2

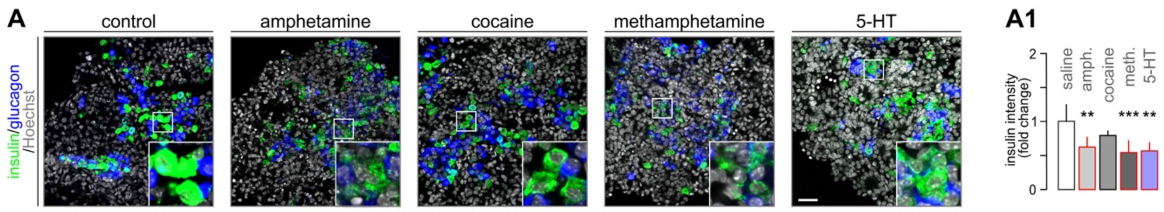

Psychostimulants decrease insulin expression in cultured embryonic pancreas. (**A**) To verify that pancreas explants (Serafimidis *et al.*, 2017) are appropriate as a cellular model of psychostimulant effects, we exposed such explants to amphetamine (10 μM), cocaine (10 μM), methamphetamine (10 μM) or 5-HT (500 nM) for 3 days *in vitro*, mimicking the *in vivo* drug-treatment paradigm. (overall drug effect by ANOVA: $F_{(4,23)} = 6.93$, $p = 0.0008$; **A1**) Not only amphetamine ($p < 0.01$) and methamphetamine ($p < 0.001$) but also exogenous 5-HT ($p < 0.001$) reduced intracellular insulin content significantly. These data suggest that disrupted 5-HT signaling (note that psychoactive drugs do not induce long-lasting 5-HT accumulation intracellularly; *data not shown*) either enhances the release of insulin or instead impairs its expression through a transcriptional program. Data were expressed as means ± s.d. *Scale bars* = 20 μm and 6 μm (*insets*).

Appendix Figure S3

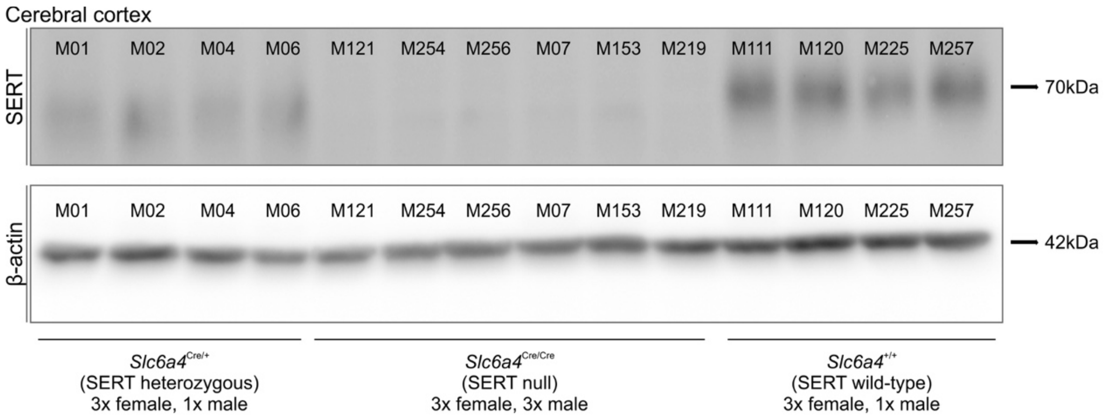

Generation of SERT (*Slc6a4*) null offspring.

We crossed *Slc6a4*$^{Cre/+}$ heterozygous knock-in mice (Zhuang *et al*., 2005) to produce phenotypically SERT null offspring. This strategy allowed us to directly compare littermates carrying different *Slc6a4* genotypes. Here, a representative Western blot on the loss of SERT protein is shown with $n \geq 4$ mice/genotype. Note that heterozygous mice (M01-M06) have halved SERT protein content in their cerebral cortex. Numbers are internal mouse identifiers. β-Actin was used as loading control. Data on SERT loss-of-function in *Slc6a4*$^{Cre/Cre}$ mice have been published elsewhere (Reisinger *et al*., 2019).

Appendix Figure S4

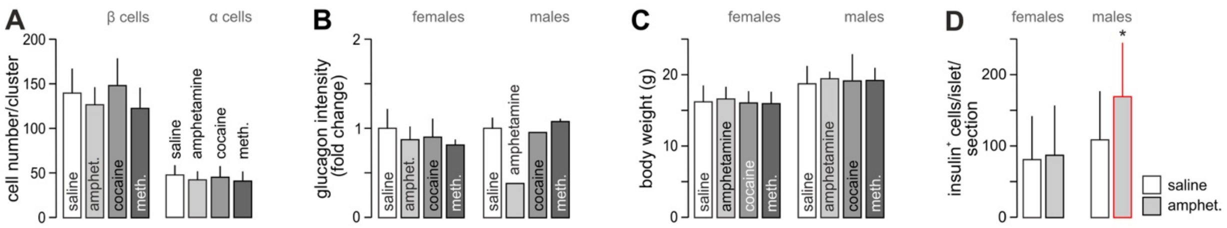

Prenatal psychostimulant exposure does not affect islet composition, glucagon content, body weight but does so for insulin^{+} β cells at 1-year of age.

(**A**) Prenatal exposure to psychostimulants did not affect β and α cell numbers in the pancreas of 6-week old mice (β cells: $F_{(3,19)} = 1.029$, $p = 0.402$; α cells: $F_{(3,19)} = 0.53$, $p = 0.668$). (**B**) Prenatal exposure to psychostimulants did not affect glucagon immunoreactivity in the pancreata of either female or male (6-week old) offspring. Data were expressed as means ± s.d. from $n \geq 3$ mice/group (except for amphetamine and cocaine in males; $n = 1$), one-way ANOVA. (**C**) Bodyweight of adult mice with *in utero* drug history. Data were expressed as means ± s.d. from $n \geq 3$ mice/group (one-way ANOVA for females: $F_{(3,23)} = 0.75$, $p = 0.533$; for males: $F_{(3,37)} = 0.199$, $p = 0.896$). (**D**) Intrauterine amphetamine exposure significantly increased the number of insulin^{+} β cells in male but not female offspring ($p < 0.05$; n = 56 mice/group; Student's *t*-test). The number of α cells in this age group did not change as a factor of either amphetamine treatment or sex.

Appendix Figure S5

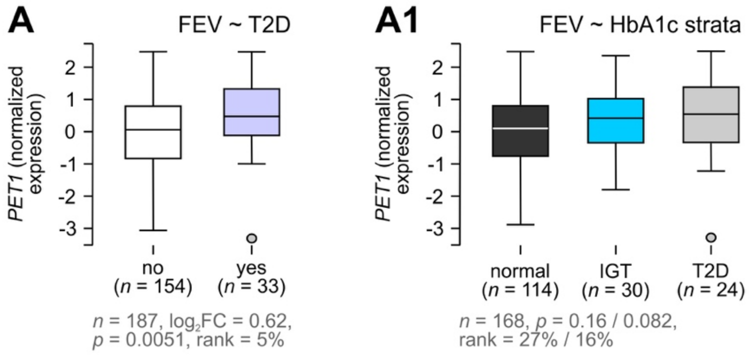

***FEV* expression and its relation to type 2 diabetes in humans.**

(**A**) Transcriptome analysis (bulk) of human pancreatic islets for *FEV* in control *vs.* patients with with type 2 diabetes (T2D). Data were expressed as log_2counts per million (CPM) and normalized to control. (**A1**) Co-expression analysis of *FEV vs.* glucose tolerance status. Samples were stratified according to glucose tolerance estimated from HbA1c, i.e. donors with normal glucose tolerance (HbA1c < 6%, *n* = 114), impaired glucose tolerance (IGT, 6% ≤ HbA1c < 6.5%, *n* = 30) and T2D (HbA1c ≥ 6.5%, *n* = 24). Single data points denote outliers. Boxes represent 25th percentiles ± 90th percentiles, horizontal lines correspond to median values.

Appendix Figure S6

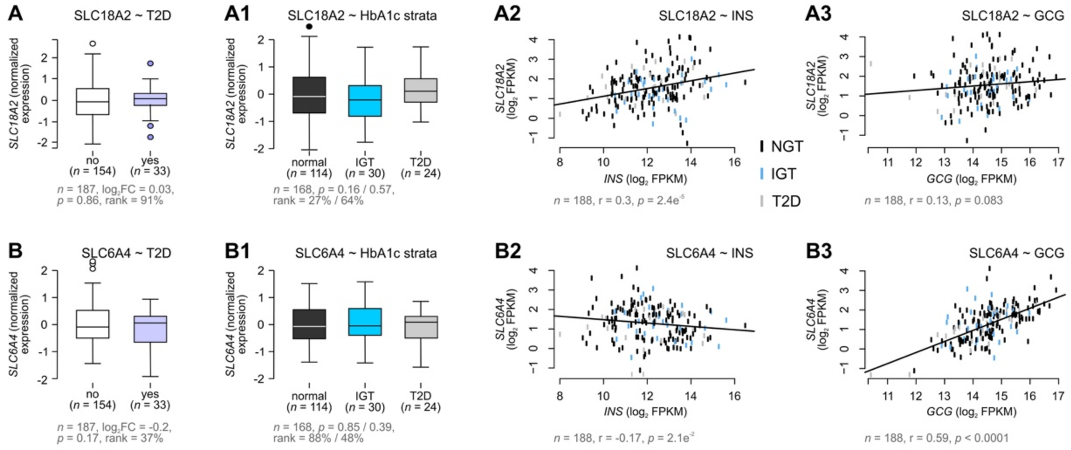

***SLC18A2* and *SLC6A4* expression and their relation to type 2 diabetes in humans.** Transcriptome analysis (bulk) of human pancreatic islets for *SLC18A2* (**A**) and *SLC6A4* (**B**) in control *vs*. patients with with type 2 diabetes (T2D). Data were presented as log_2counts per million (CPM), normalized to control. For subsequent analysis, all samples were stratified according to glucose tolerance estimated from HbA1c, i.e. donors with normal glucose tolerance (HbA1c < 6%, n = 114, black label in co-expression analysis), impaired glucose tolerance (IGT, 6% ≤ HbA1c < 6.5%, n = 30, blue label in co-expression analysis), and T2D (HbA1c ≥ 6.5%, n = 24, grey label in co-expression analysis). Co-expression analysis of *SLC18A2* (**A1**) and *SLC6A4* (**B1**) *vs*. glucose tolerance status. Single data points denote outliers. Boxes represent 25th percentiles ± 90th percentiles, horizontal lines represent median values. Co-expression analysis of *SLC18A2 vs*. *INS* (**A2**) or *GCG* (**A3**) and *SLC6A4 vs*. *INS* (**B2**) or *GCG* (**B3**).

References

Reisinger SN, Wanek T, Langer O, Pollak DD (2019) PET imaging of the mouse brain reveals a dynamic regulation of SERT density in a chronic stress model. *Transl Psychiatry* 9:80.

Serafimidis I, Rodriguez-Aznar E, Lesche M, Yoshioka K, Takuwa Y, Dahl A, Pan D, and Gavalas A (2017) Pancreas lineage allocation and specification are regulated by sphingosine-1-phosphate signalling. *PLoS Biol* 15: e2000949.

Zhuang X, Masson J, Gingrich JA, Rayport S, Hen R (2005) Targeted gene expression in dopamine and serotonin neurons of the mouse brain. *J Neurosci Methods* 143:27-32.
