# Supplementary material for: Life‐long impairment of glucose homeostasis upon prenatal exposure to psychostimulants
Source: EMBO J. 2019 Nov 21;39(1):e100882. doi: 10.15252/embj.2018100882 (PMC6939201; doi:10.15252/embj.2018100882)
Supplement: Supplementary file 2 — Expanded View Figures PDF [file EMBJ-39-e100882-s002.pdf]

Expanded View Figures

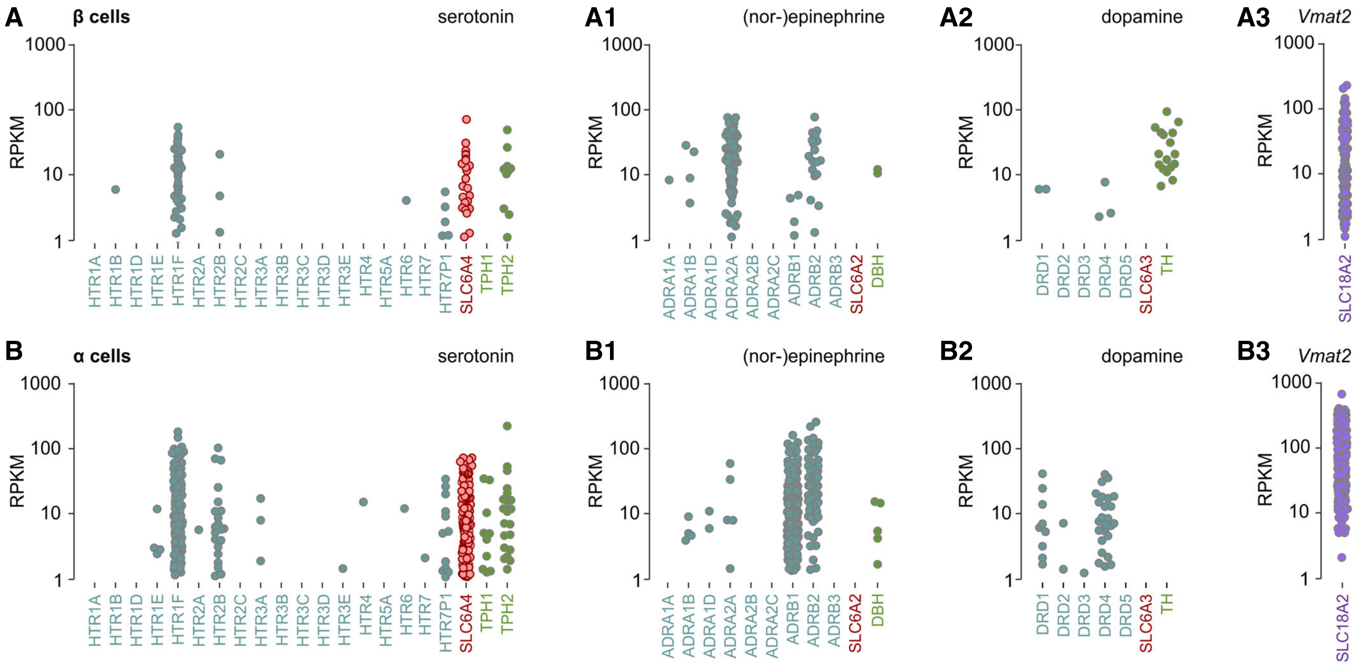

Figure EV1. Single-cell RNA-seq-based reconstruction of monoamine signaling in human pancreatic β cells.

A, B Single-cell RNA-seq analysis of β (A-A_3) and α cells (B-B_3) from healthy (n = 6) and type 2 diabetic (n = 4) donors revealed the presence (or lack) of molecular determinants for serotonin, epinephrine/norepinephrine, and dopamine signaling. Note particular differences in serotonin transporter (SLC6A4), tryptophan hydroxylase (TPH2), and tyrosine hydroxylase (TH) expression between β and α cells. Expression of the vesicular monoamine transporter (VMAT2), which is non-selective for monoamines, was plotted separately (A_3, B_3). Data were expressed as $\log_2$ reads per kilobase of transcript per million mapped reads (RPKM). Data were re-processed from an open-source database (Segerstolpe *et al*, 2016).

© 2019 The Authors

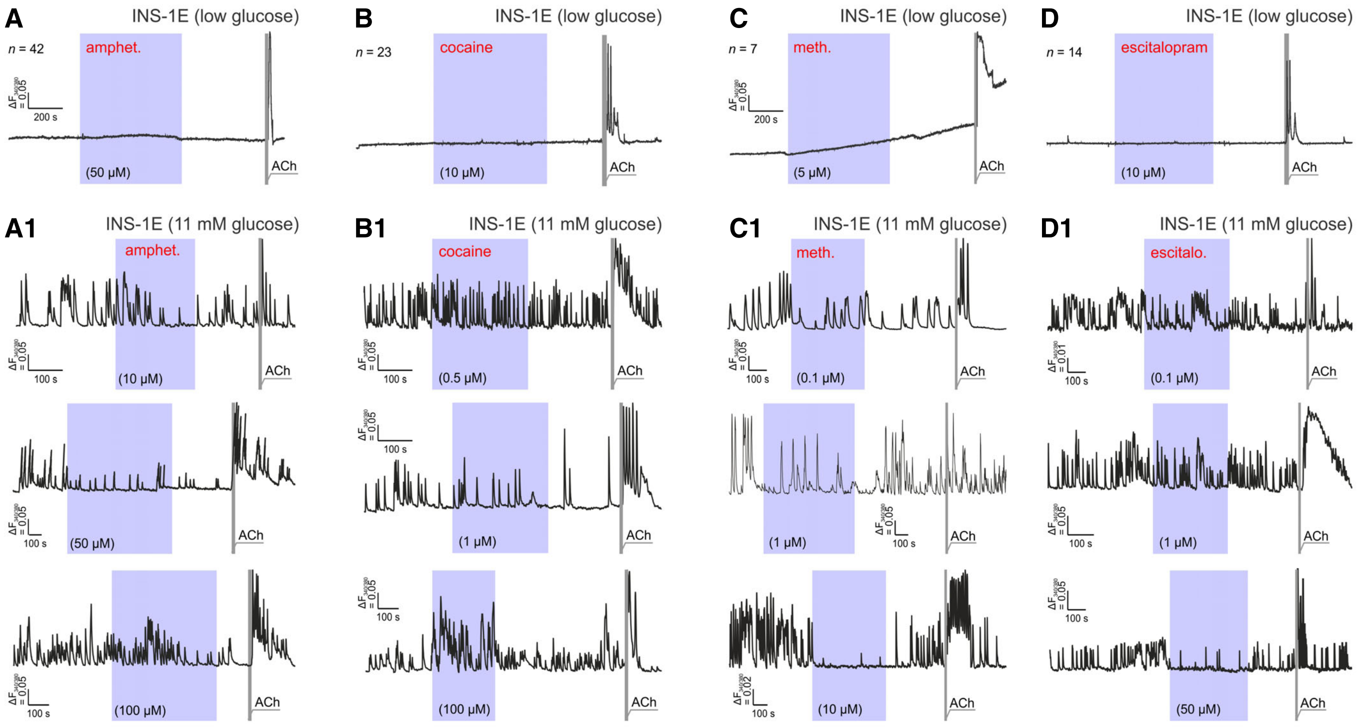

Figure EV2. Psychostimulants reduce glucose-induced Ca^{2+} oscillations in INS-1E cells.

A–D INS-1E cells do not produce spontaneous Ca^{2+} oscillations in the presence of 3 mM glucose. When applied in low glucose (3 mM)-containing medium, none of the psychostimulants affected intracellular Ca^{2+} in INS-1E cells either. The time of superfusion at specific drug concentrations is marked as shaded purple background. Acetylcholine (ACh; 5 µM) was used as positive control throughout. (A1, B1, C1, D1) Representative recordings of intracellular Ca^{2+} oscillations in 11 mM glucose with the drug concentrations indicated. All drugs were applied acutely by superfusion. Quantitative data on the amplitude and frequency of intracellular Ca^{2+} oscillations are shown in Fig 3.

© 2019 The Authors

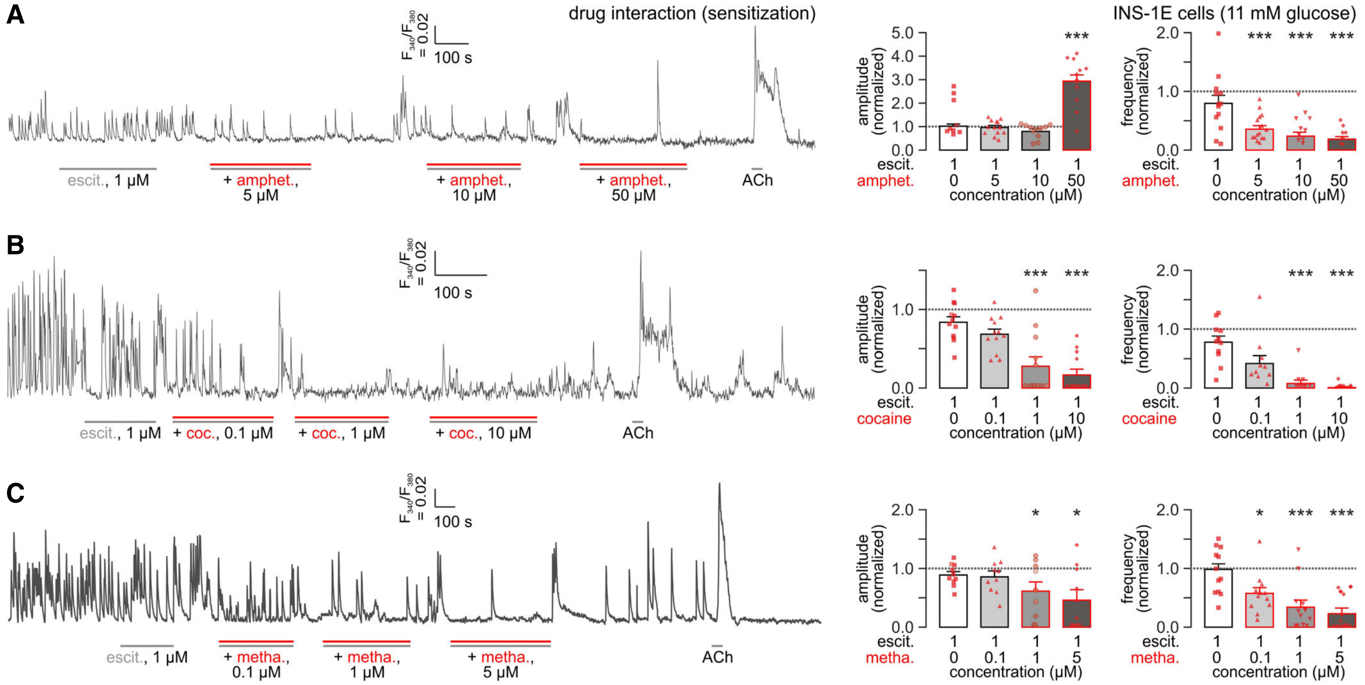

Figure EV3. Modifications of psychoactive drug effects on intracellular Ca^{2+} dynamics by escitalopram pre-treatment and competition in INS-1E cells.

A (*left*) Representative recoding of Ca^{2+} oscillations in INS-1E cells (in 11 mM glucose) exposed to escitalopram ("escit."), a selective SERT inhibitor, at a concentration that minimally affects baseline Ca^{2+} dynamics. Incrementing concentrations of amphetamine ("amphet.") were applied as indicated. (*middle*) The interaction of escitalopram and 50 µM amphetamine significantly increased the amplitude of Ca^{2+} oscillations. This was interpreted as the summation of oscillations particularly because of their reduced frequency (*right*).

B (*left*) Representative recording showing the interaction of escitalopram with increasing concentrations of cocaine ("coc.") as indicated. Note that both the amplitude (*middle*) and frequency (*right*) of intracellular Ca^{2+} oscillations were significantly and dose-dependently reduced.

C (*left*) The effect of escitalopram and methamphetamine ("metha.") alone and in combination on Ca^{2+} oscillations in INS-1E cells. A representative recording trace is shown. Escitalopram x methamphetamine also reduced both the frequency (*middle*) and amplitude (*right*) of intracellular Ca^{2+} oscillations even though methamphetamine seemed less efficacious than cocaine.

Data information: Horizontal colored lines indicate the time periods of drug superfusion. Acetylcholine (ACh; 5 µM) was used as positive control throughout. Dotted lines correspond to normalized baseline. Symbols in red denote biological replicates. Data were normalized to baseline and expressed as means ± SEM; **P* < 0.05, ****P* < 0.001 versus baseline (Student's *t*-test).

 © 2019 The Authors

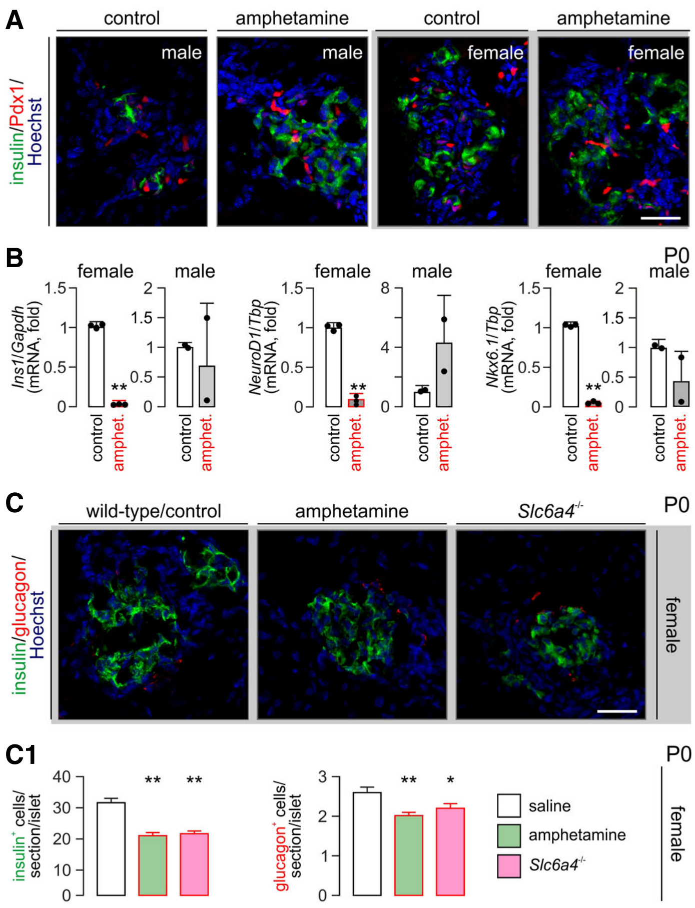

Figure EV4. Consequences of intrauterine amphetamine exposure on select transcription factors at birth and in *Scl6a4*$^{-/-}$ mice.

A No significant difference was noted in the number of histochemically detectable Pdx1^{+} cells prenatally exposed to amphetamine versus vehicle-treated controls at P0. An effect of sex was not observed either.

B Real-time PCR experiments pointed to a sex-independent reduction of *Ins1*, *NeuroD1*, and *Nkx6.1* mRNAs in pancreata from P0 mice. Note that amphetamine in all cases induced a marked reduction albeit reaching statistical significance (n = 3 animals/sex/group).

C In female offspring, *Slc6a4* knock-out seemed to phenocopy the effect of intrauterine amphetamine exposure by significantly reducing the number of insulin^{+} β cells (C1). Note that α cells were also adversely affected in this experiment. $n \geq 3$ animals/sex/group.

Data information: *Scale bar* = 40 μm (C), 25 μm (A). Data were expressed as means ± SEM. ***P* < 0.01, **P* < 0.05 (pair-wise comparisons after one-way ANOVA).

© 2019 The Authors

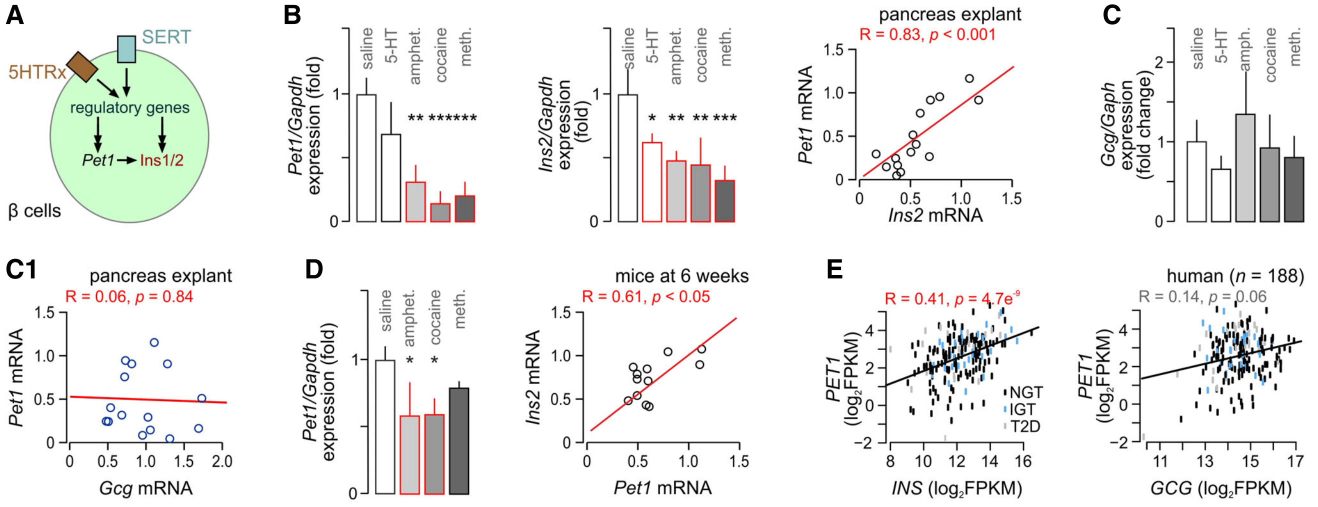

Figure EV5. Prenatal psychostimulant exposure concordantly reduces *Pet1*/*Fev* and insulin expression.

A Schematic outline of a 5-HT-dependent mechanism inducing changes in insulin (*Ins1/2* in mouse) production independently or through the transcription factor *Pet1*/*Fev*. Note that 5-HT receptors (5HTRx, non-specified subclass) could contribute by affecting regulatory genes upstream from either *Pet1*/*Fev* or *Ins1/2*, or both. Likewise, the serotonin transporter (SERT) could affect *Pet1*/*Fev* or *Ins1/2* directly or through upstream regulatory elements (indirect cascade).

B *Ex vivo* treatment with psychostimulants but 5-HT significantly decreased both *Pet1*/*Fev* (*left*) and *Ins2* (*middle*) mRNA levels in fetal pancreatic explants. In these experiments, *Pet1*/*Fev* and *Ins2* mRNA expression positively correlated (*right*).

C None of the treatments affected glucagon (*Gcg*) expression, which did not correlate with that of *Pet1*/*Fev* either (C1).

D *Pet1*/*Fev* expression (*left*) and its correlation with *Ins2* in 6-week-old offspring prenatally exposed to the psychostimulants indicated.

E Positive Spearman's correlation between PET1/FEV and INS but not GCG mRNA levels in pancreatic islets form healthy (normoglycemic; NGT in black) and diabetic donors (impaired glucose tolerance (IGT; blue) and diabetic (T2D; gray)). Data were expressed as log2 fragments per kilobase million (FPKM).

Data information: Quantitative data from $n \geq 3$ explants or mice/group were expressed as means ± SD, ***$P < 0.001$, **$P < 0.01$, *$P < 0.05$ (pair-wise comparison after one-way ANOVA).

© 2019 The Authors
