# Supplementary material for: Life‐long impairment of glucose homeostasis upon prenatal exposure to psychostimulants
Source: EMBO J. 2019 Nov 21;39(1):e100882. doi: 10.15252/embj.2018100882 (PMC6939201; doi:10.15252/embj.2018100882)
Supplement: Supplementary file 3 — Movie EV1 [file EMBJ-39-e100882-s003.zip › Legend_Movie_EV1.docx]

Movie EV1. Lightsheet microscopy saline/control P0 tissue. Three-dimensional reconstruction of new-born pancreata after prenatal amphetamine or vehicle exposure by light-sheet microscopy. Tissues were opticallycleared *en bloc* and immunolabelled for insulin. High-resolution rendering of insulin+pancreatic islets is shown in Figure 5B,B1.
