# Supplementary material for: Life‐long impairment of glucose homeostasis upon prenatal exposure to psychostimulants
Source: EMBO J. 2019 Nov 21;39(1):e100882. doi: 10.15252/embj.2018100882 (PMC6939201; doi:10.15252/embj.2018100882)
Supplement: Supplementary file 4 — Movie EV2 [file EMBJ-39-e100882-s004.zip › Legend_Movie_EV2.docx]

Movie EV2. Lightsheet microscopy P0 amphetamine. Three-dimensional reconstruction of new-born pancreata after prenatal amphetamine or vehicle exposure by light-sheet microscopy. Tissues were opticallycleared *en bloc* and immunolabelled for insulin. High-resolution rendering of insulin+pancreatic islets is shown in Figure 5B,B1.
